# Thiamine Deficiency and Neuroinflammation Are Important Contributors to Alcohol Use Disorder

**DOI:** 10.3390/pathophysiology32030034

**Published:** 2025-07-04

**Authors:** Nikhila Kalapatapu, Samantha G. Skinner, Emma G. D’Addezio, Srija Ponna, Enrique Cadenas, Daryl L. Davies

**Affiliations:** 1 Institute for Addiction Science, University of Southern California, Los Angeles, CA 90033, USA; kalapata@usc.edu; 2 Titus Family Department of Clinical Pharmacy, Alfred E. Mann School of Pharmacy and Pharmaceutical Sciences, University of Southern California, Los Angeles, CA 90033, USA; sgskinne@usc.edu (S.G.S.); daddezio@usc.edu (E.G.D.); srija.ponna@gmail.com (S.P.); 3 Department of Pharmacology and Pharmaceutical Sciences, Alfred E. Mann School of Pharmacy and Pharmaceutical Sciences, University of Southern California, Los Angeles, CA 90033, USA; cadenas@usc.edu

**Keywords:** alcohol, AUD, gut-liver-brain axis, thiamine, neuroinflammation, TLR4, NLRP3

## Abstract

Despite the growing morbidity associated with alcohol use disorder (AUD), current FDA-approved therapeutics fail to adequately address the condition. This is in part due to the complex systemic effects of ethanol (EtOH), which have particularly negative consequences on the gut–liver–brain axis. Importantly, two systemic mechanisms underlying the progression of AUD remain underemphasized in therapeutic development: thiamine deficiency and neuroinflammation. Alcohol-induced thiamine deficiency leads to reduced activity of key metabolic enzymes, thereby resulting in energy deficits, oxidative stress, and severe clinical implications. EtOH also activates TLR4 and NLRP3, both of which play critical roles in the regulation of neuroimmune responses. While research directly investigating the relationship between thiamine deficiency and neuroinflammation is still in its early stages, our review highlights the emerging connections between these two seemingly distinct pathomechanisms. Additionally, potential therapeutic approaches and targets for addressing AUD at a systemic level are discussed.

## 1. Introduction

Alcohol use disorder (AUD) is a chronic, relapsing condition classified by the Diagnostic and Statistical Manual of Mental Disorders (DSM-V) as a substance-related and addictive disorder marked primarily by problematic patterns of alcohol intake. There are eleven diagnostic criteria for the condition, including the persistent desire to drink alcohol, difficulty controlling alcohol intake, continued use despite harmful consequences, tolerance, and withdrawal in the absence of alcohol use [1]. A complex disorder, the etiopathogenesis of AUD encompasses a maladaptive activation of reward circuitry, abnormal frontal–striatal and amygdala–striatal connectivity, impaired dopamine neurotransmission, and reductions in inhibitory signaling [2,3,4].

In 2023, over 10% of Americans aged 12 and older met the specified criteria for an AUD [5]. AUD is most prevalent among non-Hispanic White individuals; however, American Indian and Alaska Native populations face the highest rates of AUD-related deaths compared to any other racial group in the United States [6,7]. While men are more frequently diagnosed with AUD, alcohol-related emergency visits, illness, and mortality are increasing at a faster rate among women [8]. Unfortunately, the rates of morbidity and mortality linked to AUD are continuing to rise, a trend associated with the coronavirus-19 pandemic. Across the United States, retail sales of beer, wine, and liquor increased by 20% between March and September of 2020 relative to the same period in 2019 [9]. Furthermore, the number of alcohol-related deaths increased by almost 38% during the first two years of the pandemic, with the largest increases occurring among those between the ages of 25 and 44 [10]. According to current estimates, over 178,000 deaths occur annually due to excessive alcohol use [11]. AUD also has a significant financial impact; the socioeconomic burden associated with excessive alcohol consumption totaled an estimated USD 249 billion in 2010, driven by a loss of workplace productivity, healthcare costs, law enforcement expenses, and motor vehicle accidents [12]. The severe societal consequences associated with AUD underscore the need for a deeper understanding of the pathological mechanisms behind the disorder.

As of 2025, there are three FDA-approved pharmacotherapies for AUD: disulfiram (Antabuse), naltrexone (Revia/Vivitrol), and acamprosate (Campral) [13]. However, despite their availability, few individuals utilize them. In 2022, only 7.6% of individuals with AUD aged 18 and older reported receiving any treatment in the past year, and only 2.2% received pharmacological treatments [14]. These low rates of treatment seeking are ascribable to several factors, including lack of therapeutic efficacy, treatment cost, poor patient education, personal beliefs, and perceived stigmas [15]. Even among those who do seek treatment, medication effectiveness remains a persistent challenge, further complicated by medication adherence, severity of alcohol use, and concomitant interventions such as behavioral therapies, support groups, and lifestyle changes. To wit, it is estimated that 60% of those seeking treatment for AUD will relapse within six months [16]. It is important to consider that the shortcomings observed with currently available treatment modalities might be attributable to weaknesses in targeting appropriate pharmacological mechanisms. Approved therapies aim to modulate drinking behaviors principally by modulating neurotransmitter systems; however, these fail to consider alternative mechanisms which drive the disease process. A comprehensive understanding of AUD pathophysiology is therefore critical to support efficacious therapeutic development.

While neurotransmitter dysregulation has long dominated the landscape of AUD research, alternative mechanisms—including metabolic and immune signaling pathways—are gaining recognition for their roles in CNS disease development and progression. In this review, we will discuss the roles of thiamine-dependent metabolic pathways and neuroinflammatory processes in relation to AUD, which to this point have not been prioritized in therapeutic development. By highlighting these mechanisms and the emerging connections between them, we hope to provide a more comprehensive understanding of alcohol use disorder and inspire new directions in pharmacological research.

## 2. Methodology

A comprehensive search of the literature was performed using PubMed and Google Scholar. The primary search terms included combinations of “thiamine deficiency”, “Wernicke-Korsakoff Syndrome”, “neuroinflammation”, “TLR4”, “NLRP3”, “alcohol use disorder”, “alcohol”, “ethanol”, “alcohol liver disease”, and “TREM2”. The search was limited to English-language articles published up to January 2025. Reference lists of key papers were also examined for additional sources. Peer-reviewed original research articles, reviews, and clinical reports that offered substantive insight into the core themes of this review were included. Both preclinical and clinical investigations were considered, provided they yielded mechanistic insights or relevant data pertinent to the scope of the review. Studies were excluded if they were not peer-reviewed or failed to meet basic standards of methodological transparency or scientific rigor. Given the narrative nature of this review, no formal quality assessment or risk of bias scoring was applied. Rather, studies were selected to represent both foundational research and emerging findings.

## 3. Organ Crosstalk in AUD

**Figure 1:**
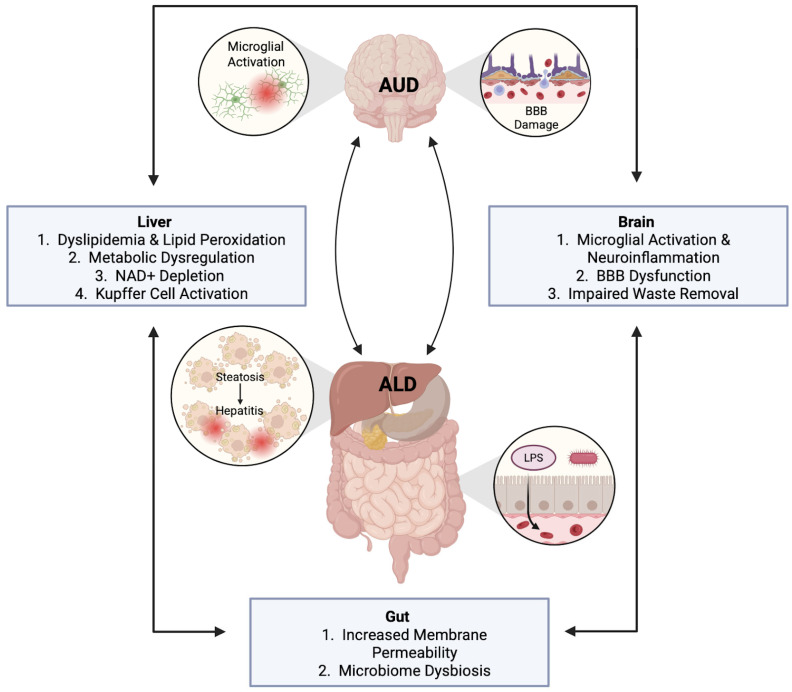
The gut–liver–brain axis and alcohol use disorder pathology. Organ crosstalk plays a critical role in AUD. Broadly, damage to the gut upon alcohol consumption leads to endotoxemia which in turn influences liver disease progression and CNS function.

Chronic alcohol consumption has a systemic impact and is associated with over 200 disease states [17]. As such, a comprehensive understanding of the biological consequences of AUD requires an examination of organ system crosstalk, the most influential example of which is the gut–liver–brain axis. Largely regulated by the gut microbiome, communication between the gut, liver, and brain via the immune and nervous systems affects a number of CNS conditions [18]. Notably, EtOH and its metabolic byproducts induce alterations to each of these organs, leading to consequential disruptions to this axis and precipitating AUD development and pathogenesis (Figure 1).

A critical point of breakdown in communication between the gut, liver, and brain occurs in the gastrointestinal tract, where EtOH compromises intestinal barrier morphology and function. Duodenal biopsies from individuals with AUD reveal significant mucosal alterations, including flattened villi, immune cell infiltration in the lamina propria, and intraepithelial lymphocytes [19]. This results in impaired nutrient absorption, chronic mucosal inflammation, and immune dysregulation. Prolonged alcohol exposure also alters the expression of critical tight junction proteins, such as claudin-1 and zonula occludens-1 (ZO-1), which weakens the mucosal barrier and leads to increased gut permeability, referred to as leaky gut [20]. Not only does EtOH damage the intestinal epithelium, but it also leads to severe gut dysbiosis. Patients with AUD possess an altered gut microbiota composition, exhibiting a shift towards Gram-negative bacteria—potent immune activators with high endotoxic activity [21,22,23]. Alcohol-associated changes to the gut microbiome in turn exacerbate increased intestinal permeability, as alcohol consumption is associated with decreased bacterial production of short chain fatty acids (SCFA), which has implications for the maintenance of the intestinal membrane barrier, as well as CNS health [24]. This disruption allows for the translocation of Gram-negative derived endotoxins into systemic circulation, as is seen in both in vivo models and AUD patients [25,26,27]. These endotoxins subsequently activate resident tissue macrophages in both the brain and the liver, triggering inflammation and oxidative stress [28,29,30].

Endotoxemia present in AUD contributes to the development of alcohol-associated liver disease (ALD), a progressive disease state attributable to chronic alcohol consumption that is marked by distinct histopathologies including steatosis, steatohepatitis, and cirrhosis. It is estimated that between 90% and 95% of patients with AUD exhibit some degree of steatosis, the initial stage of ALD characterized by the accumulation of lipid droplets in hepatocytes [31]. Much of the damage seen in ALD can be attributed to EtOH metabolism, a reactive process which disrupts metabolic homeostasis. EtOH is primarily metabolized in the liver by two enzymes, alcohol dehydrogenase (ADH) and aldehyde dehydrogenase (ALDH), ultimately leading to the production of acetate. Together, they catalyze a reductive reaction that depletes nicotinamide adenine dinucleotide (NAD+). Two minor pathways—cytochrome P450 (CYP2E1) and catalase—also contribute to EtOH metabolism, particularly during periods of heavy alcohol consumption [32]. While catalase is constitutively expressed, CYP2E1 is induced by chronic EtOH exposure and exhibits a greater metabolic capacity for ethanol oxidation than ADH, which leads to an increased generation of acetaldehyde and reactive oxygen species (ROS) [33]. Acetaldehyde, a highly reactive intermediate produced by all EtOH-metabolizing enzymes, forms DNA and protein adducts that induce mutations, disrupt protein function, and trigger pro-inflammatory responses [34,35]. In this way, EtOH metabolism drives oxidative stress and lipid peroxidation, creating an environment of metabolic dyshomeostasis that sensitizes the liver to further injury. These processes, together with the activation of hepatic Kupffer cells by endotoxins and oxidative stress, promote progression from steatosis to steatohepatitis, a cell state marked by inflammation and cell death, which can then further advance through non-reversible stages of fibrosis, cirrhosis, and ultimately hepatocellular carcinoma [36].

Chronic alcohol consumption not only disrupts peripheral organ systems but also has profound effects on the CNS. EtOH and its metabolic byproducts contribute to neurotoxicity through several mechanisms, including the depletion of NAD+. NAD+ deficiency has been linked to impaired antioxidant defense capacity, reduced ATP production, diminished neuronal function, and gene silencing in several CNS pathologies, including Alzheimer’s and Parkinson’s disease [37]. Beyond these metabolic disruptions, chronic EtOH exposure also engages neuroimmune pathways—most notably by activating microglia and initiating a sustained pro-inflammatory response [38]. Specifically, EtOH exposure promotes microglial polarization to an M1 phenotype, wherein microglia release pro-inflammatory cytokines and chemokines, ultimately contributing to neuroinflammation and oxidative stress [39]. Microglial activation reflects both the direct neurotoxic actions of ethanol and the indirect impact of systemic inflammation, including circulating endotoxins from the intestine and pro-inflammatory mediators from the liver. Additionally, a recent study found a positive association between alcohol-induced dyslipidemia and glymphatic system dysfunction in heavy drinkers with alcohol-related brain damage [40]. The glymphatic system is critical for waste removal from the brain and its dysfunction allows for the accumulation of toxic substances. Furthermore, when the liver’s detoxification abilities are severely compromised, toxins can enter the brain and result in a condition called hepatic encephalopathy (HE) [41]. HE is most commonly associated with persistent alcohol misuse and is characterized by severe cognitive, psychiatric, and motor impairments. Notably, HE is frequently diagnosed alongside Wernicke–Korsakoff syndrome, a severe neurological condition that occurs in individuals with chronic alcohol use disorder. The cumulative effect of these processes highlights the central role of metabolic dysfunction and inflammation in AUD pathogenesis and its broader impact on systemic organ crosstalk.

## 4. Implications on Thiamine-Related Metabolic Disruptions in AUD

**Figure 2:**
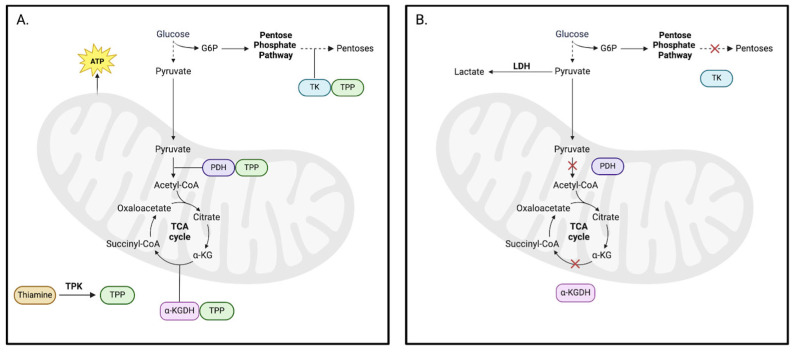
Thiamine-dependent enzymes. (**A**) Thiamine-sufficient metabolism. Bioactive thiamine, TPP, is a cofactor for three enzymes with important roles in cellular metabolism: PDH, ɑ-KGDH, and TK. In normal cellular conditions with adequate TPP levels, glucose is converted into pyruvate through the process of glycolysis. Pyruvate is then turned into acetyl-CoA, a reaction catalyzed by PDH in the mitochondria. Acetyl-CoA is oxidized in the TCA cycle, a multistep process involving the enzyme ɑ-KGDH, and is then directed to the electron transport chain for further oxidation and production of ATP. Additionally, an intermediate of glycolysis, glucose 6-phosphate (G6P), can be shunted into the pentose phosphate pathway where it is used to generate an antioxidant, NADPH, by the enzyme TK. (**B**) Thiamine-deficient metabolism. In thiamine-deficient conditions, the activation of these three enzymes is inhibited, causing impairments to cellular metabolism (denoted by red X). The cell must then adapt to an alternate form of cellular respiration through the production of lactate via LDH.

Thiamine (vitamin B1) is an essential dietary component that plays a crucial role in energy metabolism, nervous system functions, cardiovascular activity, and digestion. The bioactive form of thiamine, thiamine pyrophosphate (TPP), is a necessary cofactor for three metabolic enzymes: transketolase (TK), pyruvate dehydrogenase (PDH), and alpha-ketoglutarate dehydrogenase (ɑ-KGDH) (Figure 2A) [42]. TK catalyzes the transfer of two carbon units in the pentose phosphate pathway (PPP), leading to the production of pentoses, which are necessary for the synthesis of nucleic acids [43]. In this way, TK is also critical to the maintenance of cellular redox through the generation of NADPH—a cofactor used in the synthesis of steroids, fatty acids, and certain neurotransmitters [44]. PDH and ɑ-KGDH are particularly vital for cellular respiration, producing energy intermediates essential for ATP production and metabolism at large. These enzymes participate in the tricarboxylic acid (TCA) cycle, which involves the conversion of pyruvate into acetyl-CoA and alpha-ketoglutarate (ɑ-KG) into succinyl-CoA. Both steps lead to the reduction of NAD+ to NADH and broadly contribute to energy production. Within the CNS, PDH also plays a central role in acetylcholine synthesis by regulating acetyl-CoA availability [42]. Similarly, ɑ-KGDH indirectly participates in glutamate and GABA synthesis by affecting the availability of ɑ-KG [42]. Impaired activity of any of these enzymes due to thiamine deficiency can consequently lead to metabolic deficits and cell damage (Figure 2B).

While there are a number of risk factors for thiamine deficiency (TD), the largest by far is excessive alcohol consumption [45]. The association between alcohol consumption and TD has been well documented, reportedly affecting up to 80% of individuals with AUD [46,47]. Various mechanisms for alcohol-related TD have been proposed, including decreased intestinal absorption and cellular uptake of thiamine, inadequate nutritional intake, and impaired thiamine phosphorylation [42,48,49]. Much of the mechanistic understanding of alcohol-related TD is derived from preclinical models, offering valuable molecular insights into thiamine signaling and its downstream consequences. Chronic alcohol consumption in rats has been shown to lead to significant inhibition of intestinal thiamine transporter 1 (THTR-1)—but not thiamine transporter 2 (THTR-2)—expression at the mRNA level, suggesting that alcohol-induced impairment of intestinal thiamine absorption is mediated by a transcriptional mechanism targeting THTR-1 [50]. In high alcohol-preferring mice; however, reduced expression of the gene encoding THTR-2, but not THTR-1, was reported in the CNS, suggesting site- and species-specific modulation of thiamine transport [51]. Additionally, thiamine diphosphokinase (TPK), the enzyme responsible for converting thiamine to its bioactive form TPP, shows markedly reduced activity in rodent models of alcohol exposure, declining by nearly 70% after acute exposure and by 50% with chronic exposure [42,52]. Impaired thiamine phosphorylation has also been demonstrated in AUD patients, who have lower TPP levels than non-AUD patients [42]. As a result, even when thiamine is present, its functional utility is severely impaired without adequate TPK activity. Taken together, this evidence serves to underscore the fundamental relationship between thiamine and alcohol consumption.

Building on this, preclinical studies have shown that alcohol-related disruptions in thiamine extend beyond transport and activation, leading to widespread structural CNS consequences. To explore these effects in rodent models, a synthetic analog of thiamine, pyrithiamine, can be administered to inhibit thiamine metabolism. Studies using this compound in conjunction with EtOH administration have found several additive effects between TD and AUD. In mice, five days of combined EtOH and pyrithiamine treatment led to markedly augmented thalamic microglial activation, pro-inflammatory gene induction, and degeneration compared to five days of pyrithiamine treatment alone [53]. In rats, combining EtOH with pyrithiamine exacerbated neurostructural changes compared to either treatment alone, leading to greater myelin thinning and small fiber quantity in the corpus callosum, greater suppression of myelin-related genes in the parietal cortex, and increased cerebellar cytotoxicity [54,55,56]. Furthermore, combined pyrithiamine treatment and diet-deficiency in rats revealed that TD leads to impairments in the blood–brain barrier (BBB) [57]. These results were corroborated in a more recent study that found elevated neuroinflammatory markers, increased BBB permeability, and neuronal death in mice receiving both pyrithiamine and a thiamine-deficient diet [58].

Taken together, the neurobiological changes observed in preclinical models of thiamine deficiency are numerous and may form a mechanistic basis for the behavioral and cognitive impairments observed in AUD populations. Thus, understanding the mechanisms behind thiamine’s deficiencies and functional impairments in response to alcohol is critical to understanding the evolution of AUD. This section will discuss the consequences of impaired cellular metabolism and thiamine deficiency on the development and progression of AUD.

### 4.1. Thiamine-Related Energy Deficits

TD has a number of downstream effects on cellular energetics mediated by thiamine-dependent enzymes. For instance, animal models have demonstrated that TD results in a 35–40% decrease in the metabolic flow of pyruvate through PDH [59]. Reductions in PDH activity can result in decreased ATP concentrations, increased reduction of pyruvate to lactate, and cell death [60,61]. Mechanistically, when PDH is compromised, pyruvate cannot be converted into acetyl-CoA, thus disrupting the initiation of the TCA cycle and leading to insufficient ATP production through the electron transport chain and oxidative phosphorylation. Such changes imply a disruption of primary metabolic processes and a shift towards anaerobic respiration, which can eventually lead to lactic acidosis [61].

Due to its significant energy dependence, the CNS appears to be most impacted by disruptions in glucose metabolism via PDH activity [62]. Impaired glucose metabolism can lead to issues related to neuronal function, ultimately resulting in tissue injury and degeneration that predominantly occurs in brain regions such as the thalamus and cerebellum, which appear to be more vulnerable to TD [63,64]. Mice with brain-specific reductions in PDH activity exhibited decreases in both the cerebral TCA cycle and glutamatergic activity, indicating that aberrant excitatory neurotransmission occurs as a consequence of reduced cerebral glucose metabolism [65]. In AUD patients with either alcohol-related cognitive impairment (ARCI) or Wernicke’s encephalopathy (WE), 18F-fluorodeoxyglucose positron emission tomography (18FDG-PET) imaging revealed that reductions in metabolic activity were most pronounced in the prefrontal medial cortex, prefrontal lateral cortex, and anterior cingulate cortex, all key regions in higher-order brain processing [66]. Another study that also examined cerebral metabolism in AUD patients identified hypermetabolism in the cerebellum but hypometabolism in the precentral, superior frontal, and parietal cortices, patterns which are related to ataxia and working memory deficits [67]. Interestingly, cerebral hypometabolism has been implicated in the cognitive deficits that are associated with substance use disorders (SUDs) [68].

TD leads to a reduction in the activity of all three thiamine-dependent enzymes, but the extent to which they are impaired appears to be enzyme-specific. Evidence suggests that TK is most sensitive to reductions in thiamine levels, with activity reductions of 60–65% observed in thiamine-deficient rats [69]. This specificity is also observed in AUD patients. Postmortem analysis of the brains of AUD patients reveals notable reductions in TK activity in the cerebellum, thalamus, frontal cortex, temporal lobe, and prefrontal cortex; meanwhile, PDH activity was selectively reduced in the prefrontal cortex and ɑ-KGDH activity was within normal limits in all brain regions [70]. However, this pattern changes with increasing TD severity. In a study examining autopsied cerebellar samples from AUD patients with Wernicke–Korsakoff syndrome (WKS), an 86.3% decrease was observed in PDH activity, a 71.3% decrease was observed in TK activity, and a 97% decrease was observed in ɑ-KGDH activity [71]. Interestingly, no significant differences were observed in enzymatic activity between controls and AUD patients without WKS. It is important to note that the differences in enzymatic disruptions observed across the aforementioned studies may also be attributable to methodological heterogeneity. Overall, while there is a clear association between alcohol-induced TD and energy deficits, further research remains to be conducted to further characterize the effect of alcohol on thiamine-dependent enzymes.

### 4.2. Thiamine-Related Redox Deficits

TD is heavily implicated in oxidative stress, a physiological imbalance between the production of ROS and the antioxidant mechanisms responsible for their detoxification. One major mechanism for this is impaired TK activity. In thiamine-deficient conditions, TK cannot fulfill its role in the PPP, leading to a decrease in NADPH and, consequently, a cellular state of oxidative stress. Administration of a thiamine-depleted diet in mice significantly reduced TK activity in the cortex and hippocampus, and decreased whole brain and hippocampal NADPH levels, after 14 days [72]. These findings suggest diminished NADP+ reduction to NADPH as a consequence of decreased TK activity in the PPP.

**Figure 3:**
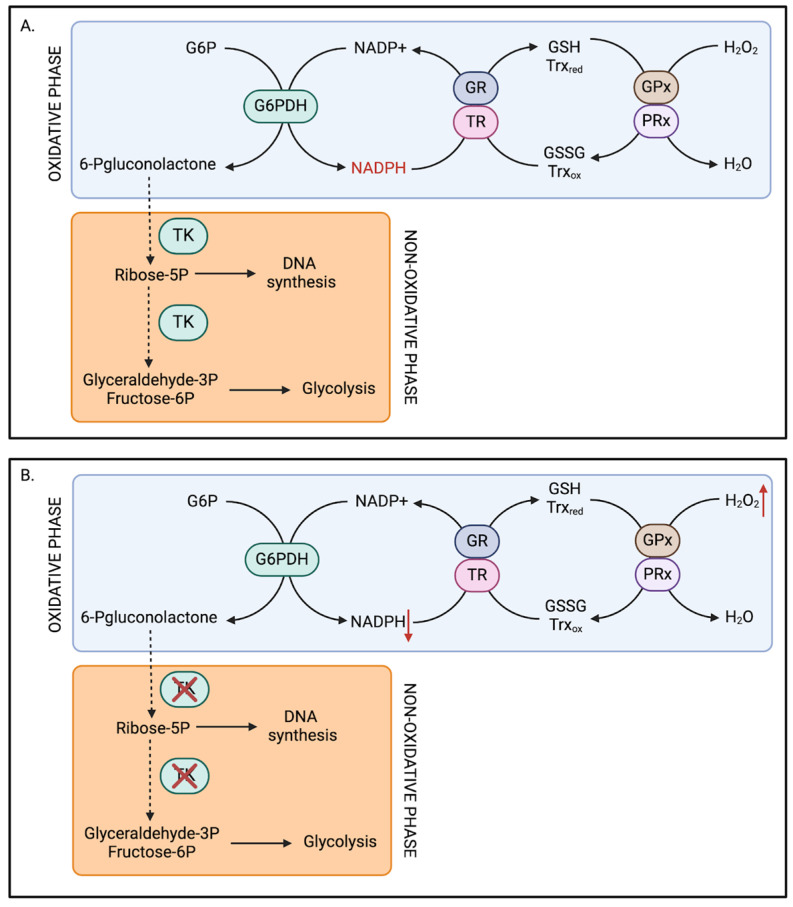
The role of thiamine in the pentose phosphate pathway. (**A**) Thiamine-sufficient redox. In the oxidative phase, G6P is oxidized into 6-phosphogluconolactone via the enzyme G6PDH, subsequently producing NADPH. 6-phosphogluconolactone transitions into the non-oxidative phase where it is metabolized to ribose-5-phosphate by transketolases (TK) and can either be used in DNA synthesis as a ribose sugar or further metabolized to glyceraldehyde-3P and fructose-6P to be used in glycolysis. Additionally, NADPH can be oxidized by glutathione reductase (GR) or thioredoxin reductase (TR) to regenerate glutathione (GSH) and thioredoxin in its reduced form, Trx_RED_. GSH and Trx_RED_ are the electron donors for glutathione peroxidase (GPx) and peroxiredoxin (PRx), leading to the reduction of hydrogen peroxide (H_2_O_2_) to H_2_O. (**B**) Thiamine-deficient redox. In thiamine-deficient conditions, reactions requiring TK activity are inhibited, preventing DNA synthesis and branching to glycolysis. NADPH production is therefore decreased (see red arrow). Upstream reactions from TKs are also inhibited, leading to the accumulation of H_2_O_2_ (see red arrow) and oxidative stress.

The decrease in NADPH in TD conditions has potentially severe consequences due to its crucial antioxidant capacity. NADPH serves as an electron donor in both the glutathione and thioredoxin pathways, catalyzing the reduction of glutathione disulfide (GSSG) to glutathione (GSH) and of thioredoxin to its reduced form (Figure 3A). Both GSH and reduced thioredoxin combat oxidative stress and have important roles in protein synthesis in the endoplasmic reticulum [73,74]. Hence, decreased NADPH levels secondary to TD sets the ground for a predominantly oxidative environment (oxidative stress) by decreasing the cofactors required by antioxidant enzymes (glutathione peroxidases and peroxiredoxins) (Figure 3B). Indeed, significant decreases in the activity of various antioxidant enzymes—including superoxide dismutase, catalase, and glutathione peroxidase—have been demonstrated in TD-treated mice [75]. However, the modulation of these antioxidant enzymes might be site-specific. For instance, in another study, while TD led to a significant increase in ROS, particularly in the thalamus and cortex, no significant changes in GSH were observed in these regions [76]. While TD may lead to an increase in ROS without affecting GSH concentrations in specific brain regions, EtOH administration induces a concentration-dependent decrease in GSH in isolated rat hepatocytes [77]. Altogether, current data emphasize the role of thiamine in the cellular redox status and suggests potential mechanisms for the associated damages seen in AUD.

### 4.3. Clinical Implications of Alcohol-Induced Thiamine Deficiency

The cellular damage induced by impaired thiamine metabolism has a number of clinical implications, ranging from mild neurocognitive impairment to more severe conditions such as Wernicke’s encephalopathy: a neurological condition marked by considerably altered mental status and ocular motor symptoms [78]. Rodent models of alcohol misuse exhibit many comparable neurocognitive deficits to those observed in AUD patients and therefore have high translational value in exploring the cognitive dysfunction associated with alcohol-induced thiamine deficiency. As is evidenced by behavioral assays performed in rats, the combination of EtOH exposure and TD has a significant impact on learning and reference memory [79]. Furthermore, these synergistic effects lead to significant impairments in cognitive flexibility and spatial memory, as well as reductions in markers of brain plasticity [80]. The relationship between cognitive deficits and TD is also clear in AUD patients, as is demonstrated by the beneficial effects of thiamine supplementation. In individuals with AUD undergoing inpatient alcohol withdrawal treatment, thiamine supplementation was found to improve cognitive function [81]. Additionally, there is an evident therapeutic relationship between thiamine supplementation dosage and working memory performance; the greatest improvements in working memory were observed in recovering AUD patients who received the highest dose of thiamine [82].

When the cognitive and neurostructural defects associated with TD become so severe, patients may develop a serious condition called Wernicke–Korsakoff syndrome (WKS). The lifetime prevalence of WKS is estimated to be 1–2% in the general population but 12–14% among individuals with AUD [83]. WKS encompasses two distinct neurological conditions: Wernicke’s encephalopathy (WE) and Korsakoff syndrome (KS). Pathologically, WE is marked by abnormal CNS physiology, specifically bilateral lesions to areas such as the periventricular nuclei, thalamus, and Papez’s circuit [84]. These lesions develop into more permanent damage if the disorder continues to progress, leading to severe cognitive dysfunction and WKS. Therefore, while WE is acute in nature, KS is a chronic and often irreversible condition with characteristic symptoms of retrograde and anterograde amnesia [85]. KS develops in approximately 80% of WE patients who fail to receive timely thiamine supplementation [85]. Notably, postmortem analysis of AUD patients with WKS reveals significant reductions in TPP-dependent enzymes, further reiterating the role of metabolism in disease progression [71].

In summary, many of the metabolic deficits exhibited in AUD can be explained by TD, which is partially responsible for the neurological changes that accompany chronic alcohol use. Impairments in thiamine-dependent enzymes have a number of negative downstream effects related to mitochondrial energetics, as is observed in the CNS and systemic health.

## 5. The Role of Neuroinflammation in Alcohol Use Disorder

Energy crises and oxidative stress represent just one group of factors contributing to the development and progression of AUD; the causative relationship between alcohol consumption and neuroinflammation has also been well established. EtOH is a known immune modulator, affecting the activity of both the innate and adaptive immune systems in several ways [86]. Insults to the immune system can be transient, particularly in the context of binge drinking, or more sustained, leading to systemic inflammation and organ damage [87,88]. There is particular interest in how chronic alcohol consumption disrupts the innate immune system in AUD, and several studies have been conducted to further this understanding. To this end, genome-wide association studies (GWASs) in individuals with AUD reveal a robust enrichment of pathways and network modules associated with immune response and inflammation [89]. Additionally, meta-analyses exploring the cytokine profiles of AUD patients relative to healthy controls reveal abnormal circulating patterns, citing strong increases in pro-inflammatory cytokines including interleukin (IL)-6, IL-7, IL-8, and tumor necrosis factor alpha (TNF-α) [90,91].

The pro-inflammatory response observed in AUD patients is largely driven by two crucial components of the innate immune system: toll-like receptor 4 (TLR4) and nucleotide-binding oligomerization domain (NOD)-like receptor (NLR) family pyrin domain-containing protein 3 (NLRP3). These proteins play critical roles in modulating systemic inflammation via the activation of pro-inflammatory signaling pathways and inflammasome assembly. Activation of these molecules in response to EtOH has been well documented. As previously discussed, damage to the integrity of the intestinal barrier by EtOH prompts the translocation of microbial products into circulation, where they may enter the liver. There, these products engage with TLR4 receptors and activate the NLRP3 inflammasome cascade [92]. NLRP3 activation in turn has been shown to promote fatty acid synthesis, contributing to the development of hepatic steatosis [93]. The activation of these proteins also has significant implications for EtOH-induced neuroinflammation, affecting AUD pathology and consummatory behaviors. As such, this section will discuss how TLR4 and NLRP3 signaling specifically contribute to the neuroimmune adaptations that underlie AUD.

### 5.1. TLR4

One class of molecule that plays a significant role in the innate immune system are toll-like receptors (TLRs). As a pattern recognition receptor (PRR), TLRs function by recognizing both pathogen-associated molecular patterns (PAMPs) and damage-associated molecular patterns (DAMPs), which stimulate downstream signal transduction pathways, ultimately leading to the expression of pro-inflammatory genes [94]. Structurally, individual TLRs consist of an ectodomain featuring leucine-rich repeats (LRRs), which facilitate the recognition of PAMPs and DAMPs, a transmembrane domain, and a cytoplasmic toll/interleukin-1 receptor (TIR) domain that mediates downstream signaling through interaction with specific adaptor proteins [94]. While there are 10 isoforms of TLR in humans, TLR4 is most commonly implicated in alcohol consumption [95]. This is largely due to two TLR4 activation signals that are released in response to EtOH: lipopolysaccharide (LPS), a PAMP that is produced by Gram-negative bacteria in the gut microbiome, and high-mobility group protein 1 (HMGB1), a DNA-binding protein that takes on the phenotype of a DAMP when in the extracellular space [96,97,98].

**Figure 4:**
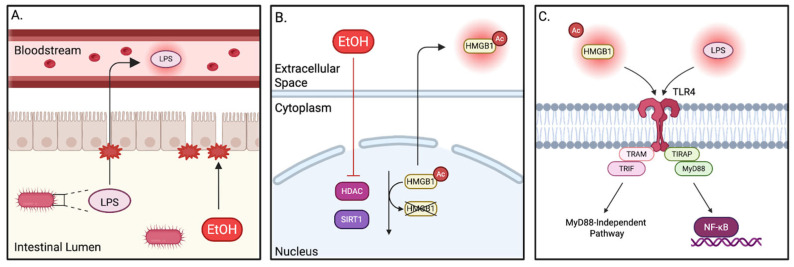
Mechanisms of alcohol-induced TLR4 activation. (**A**) increased membrane permeability secondary to ethanol consumption. EtOH disrupts tight junction proteins essential to intestinal barrier integrity, leading to increased gut permeability and allowing LPS to enter the bloodstream. (**B**) Inhibition of histone deacetylation secondary to ethanol consumption. HDAC and SIRT1 are enzymes that remove acetyl groups from both histone proteins and certain non-histone proteins such as HMGB1. This process of deacetylation, which occurs in the nucleus of the cell, is inhibited by the presence of extracellular EtOH, leading to increased levels of acetylated HMGB1. Acetylated HMGB1 is then actively secreted into the extracellular space. (**C**) Inflammatory response stimulated by DAMPs and PAMPs. Elevated concentrations of DAMPs and PAMPs stimulate TLR4 receptors, activating an inflammatory response through the MyD88-dependent or -independent pathway. The MyD88-dependent pathway involves the sequential recruitment of TIRAP followed by MyD88, resulting in the production of pro-inflammatory mediators through NF-κB upregulation. Conversely, the MyD88-independent pathway relies on the recruitment of TRAM and TRIF adaptor proteins.

Numerous studies in animal models and humans have demonstrated that both LPS and HMGB1 are upregulated following chronic alcohol exposure [27,99,100,101,102,103]. As previously discussed, chronic alcohol consumption often leads to compromised gut barrier function, enabling the translocation of LPS from the gut lumen into the bloodstream (Figure 4A) [104]. HMGB1 translocates from the nucleus in response to hyperacetylation, which can occur via multiple pathways. EtOH exposure has been shown to reduce expression of histone deacetylase (HDAC), a regulatory enzyme that removes acetyl groups from proteins, thereby allowing for the hyperacetylation of HMGB1 (Figure 4B) [105]. Sirtuin 1 (SIRT1), another histone deacetylase that acts on HMGB1, is NAD+ dependent and is therefore similarly diminished by alcohol metabolism [98,106,107]. Both of these mechanisms can be activated by the overconsumption of alcohol, thereby permitting the secretion of HMGB1.

Binding of HMGB1 or LPS induces a conformational change in the TLR4 receptor complex, enabling the recruitment of myeloid differentiation primary response gene 88 (MyD88) [108]. This is facilitated by the bridging adaptor protein, TIR-domain-containing adaptor protein (TIRAP). MyD88 recruitment to TLR4 leads to the activation of nuclear factor-κB (NF-κB), a transcription factor that is responsible for the production of many pro-inflammatory cytokines and chemokines [109]. TLR4 can also exert its effects through a MyD88-independent pathway, which involves TIR domain-containing adaptor-inducing interferon-β (TRIF) and TRIF-related adaptor molecule (TRAM) (Figure 4C) [110].

TLR4 signaling is essential to the pathogenesis of neuroinflammation in AUD. TLR4 knockout (KO) mouse models have consistently demonstrated that TLR4-deficiency is protective against alcohol-induced microglial activation, pro-inflammatory cytokine release, and apoptosis [111,112,113,114]. The role of TLR4 in EtOH-induced inflammation has also been demonstrated with treatments that disrupt its signaling pathway. For instance, use of TLR- or IL-1R-neutralizing antibodies in EtOH-treated rat cortical astrocytes reduced the immunomodulatory effects of EtOH and inhibited cell death [115]. Furthermore, administration of the anti-inflammatory molecule oleoylethanolamide to rats prior to binge EtOH exposure prevented HMGB1 and TLR4 expression in the frontal cortex and arrested the NF-κB signaling pathway [102]. Numerous additional studies have reported that elimination of TLR4 not only prevents alcohol-induced neuroinflammation, but also alcohol-related behavioral and cognitive dysfunctions [116,117,118]. Taken together, there is ample evidence of TLR4-mediated neuroinflammation following alcohol consumption.

TLR4 also appears to modulate neuroinflammation through its effects on CNS health and structural integrity. TLR4 has begun to emerge as a potential mediator of alcohol-induced neuronal injury and BBB impairment [119]. Regarding neuronal health, KO of TLR4 abrogated EtOH-induced synaptic and myelin disturbances [117,120,121]. Additionally, TLR4 signaling has been associated with increased BBB permeability in both humans and animal models after prolonged EtOH exposure [119,122]. As a result of alcohol-induced BBB disruption, circulating immune cells are able to migrate to the CNS and further perpetuate neuroinflammation (Figure 2B) [110].

The role of TLR4 in alcohol consumption behaviors and resultant neuroinflammation may be influenced by interactions with other receptors. For instance, several studies suggest that TLR4 interacts with the α2 subunit of GABAA receptors and, through this engagement, fosters alcohol preference and binge drinking in animal models [123,124,125]. Additionally, various TLRs seem to function in tandem with TLR4 to promote alcohol-induced neuroinflammation. In particular, both TLR2 and TLR9 upregulation are observed in conjunction with increased TLR4 expression in alcohol-fed mice [126,127]. In fact, the dual stimulation of TLR2 or TLR9 with TLR4 resulted in a greater inflammatory response relative to TLR4 activation alone, implying that these TLRs amplify TLR4 signaling [128]. Despite these findings, the precise mechanism by which alternative TLRs contribute to canonical TLR4 signaling remains unclear. For instance, analysis of alcohol intake in mice found that TLR4 KO had no effect on consumption behavior, while TLR2 KO decreased alcohol intake and MyD88 KO increased intake [129].

It is important to acknowledge that systemic alcohol-induced immune modulation also has direct implications for neuroinflammation. Chronic alcohol consumption enhances TLR4 signaling across multiple organ systems, contributing to a pro-inflammatory state that can affect the CNS through peripheral-to-central immune crosstalk. For example, prolonged alcohol administration significantly amplified TLR4-induced TNF-α expression in human monocytes, directly contrasting observations made in acute administration settings [130]. Additionally, mice exposed to alcohol for ten days showed elevated liver mRNA expression of several TLRs, including TLR4, TLR2, and TLR9 [131]. The same group also exhibited increased TNF-α mRNA expression in the liver, indicating a heightened sensitivity of TLRs to their associated ligands. Upon release of TNF-α and other circulating pro-inflammatory cytokines into circulation, they pass through the BBB and impact CNS signaling.

### 5.2. NLRP3

**Figure 5:**
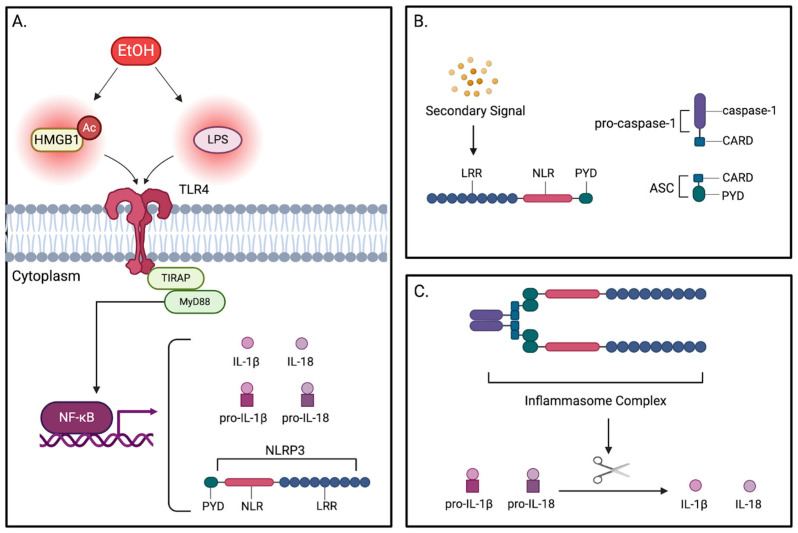
Mechanism of NLRP3 inflammasome activation. (**A**) Inflammasome priming. In response to alcohol, PAMPs and DAMPs (LPS and acetylated HMGB1) activate TLR4. TLR4 subsequently binds to TIRAP, thereby recruiting the MyD88 adaptor protein. Recruitment of MyD88 activates the transcriptional factor NF-kβ, stimulating expression of the pro-inflammatory cytokines, pro-IL-1β and pro-IL-18. NLRP3, made up of the PYD, NLR, and LRR domains, is also expressed, completing the priming step of the inflammatory pathway. (**B**) Inflammasome activation. NLRP3 activation via a secondary signal induces a conformational change allowing for oligomerization into the larger inflammasome complex; recognition of secondary signals occurs at the LRR domain of NLRP3. (**C**) Inflammasome Assembly. ASC and pro-caspase-1 are recruited, binding one another at their respective CARD regions. Likewise, the PYD region of ASC binds to the PYD region of NLRP3. Upon assembly of the complex, pro-caspase-1 is activated into caspase-1 by the process of autoproteolysis. Capase-1 then cleaves pro-IL-1β and pro-IL-18 into their active forms, IL-1β and IL-18.

Another family of PRRs that holds a crucial role in the innate immune system are nucleotide-binding oligomerization domain (NOD)-like receptor (NLR) family pyrin domain-containing proteins (NLRPs). Of the 22 documented NLRPs, NLRP3 is of particular translational interest due to its ability to form inflammasomes. These multi-protein complexes consist of an adaptor protein, apoptosis-associated speck-like protein containing a CARD domain (ASC); an effector protein, pro-caspase-1; and a sensor protein [132]. Currently, only five PRRs have been characterized for their role as a sensor protein in the inflammasome complex: NLRP1, NLRP3, NLRC4, absent-in-melanoma 2 (AIM2), and pyrin [133,134]. NLRP3 is composed of three main domains: the NLR domain, which is responsible for oligomerization of the inflammasome complex; the LRR domain, which senses ROS produced by DAMPs and PAMPs; and the pyrin domain (PYD), which facilitates protein–protein interactions between NLRP3 and ASC [135]. Canonical NLRP3 inflammasome activation is understood to be governed by two distinct signals: a priming signal (NF-κB activation) and an activation signal, which encompasses a diverse array of agonists including ROS, lysosomal damage, and extracellular ATP [136,137]. Often, in the context of EtOH consumption, the priming signaling for the molecular activation of NLRP3 is mediated by TLR4 signaling, while secondary signals including ROS subsequently drive inflammasome activation (Figure 5A,B).

Once expressed by the NF-κB transcriptional pathway, NLRP3 is activated by a secondary signal, prompting assembly of the inflammasome complex. Upon assembly, pro-caspase-1 undergoes cleavage into its active form, caspase-1, through the process of autoproteolysis [136,138]. Caspase-1 is then able to cleave pro-IL-1β and pro-IL-18 into their respective bioactive forms, IL-1β and IL-18. Additionally, caspase-1 activation can also trigger pyroptosis of the cell (Figure 5C) [137,139].

The role of NLRP3 in alcohol-induced inflammation and injury has been well characterized. In aged mice exposed to an intermittent binge EtOH schedule, researchers observed notable increases in the expression of pro-inflammatory mediators (TNF-α, IL-1β, CCL2), enhanced activation of the NLRP3 inflammasome, and increased microglial reactivity [140]. Evidence also suggests that repeated alcohol exposure hyperactivates the NLRP3 inflammasome, demonstrated by the hypersecretion of IL-1β by human and murine leukocytes exposed to EtOH [141]. Hypersecretion of IL-1β was found to be dependent upon mitochondrial ROS production and abrogated by ADH inhibition. Notably, the activation of NLRP3 by EtOH promotes cell death by pyroptosis and apoptosis, further contributing to neuroinflammation and brain damage [142]. As TLR4 signaling is heavily implicated in NLRP3 inflammasome priming, the two immune receptors are often linked in empirical research. TLR4 deletion prevents most EtOH-induced NLRP3 activation and reduces cell death, illustrating the role of TLR4/NLRP3 crosstalk in EtOH-induced damage [142]. Consistent with evidence of BBB disturbances in response to TLR4 activation, chronic EtOH treatment heightens microglial activation by stimulating NLRP3 signaling; this in turn increases BBB permeability and allows for leukocyte infiltration, exacerbating neuroinflammation and brain damage [143]. TLR4 suppression eliminates most NLRP3-dependent microglial reactivity, disruptions to BBB integrity, and leukocyte entry into the brain.

NLRP3 signaling is also suspected to play a role in alcohol consumption behaviors. Alcohol consumption and preference were significantly reduced in mice following pharmacological inhibition of NLRP3. Interestingly, these behavioral effects were sex-dependent: in female mice, inhibition of both NLRP3 and caspase-1 significantly reduced alcohol intake and preference, whereas in male mice, only NLRP3 inhibition reduced alcohol consumption, and caspase-1 inhibition had no effect [144]. Mechanistic studies demonstrate that NLRP3 elimination in mice appears to decrease binge alcohol intake and anxiety-like behaviors by reducing glutamatergic neurotransmission in corticostriatal circuits [145]. Glutamate has been implicated in relapse triggered by cues or alcohol exposure, and as such is believed to play a critical role in alcohol dependence [146].

Studies suggest that while acute alcohol exposure targets inflammasome activity, chronic exposure affects both activity and assembly. In an acute setting, EtOH pre-treatment of LPS-primed human macrophages suppressed the release of IL-1β in a dose-dependent manner and reduced caspase-1 activation, but did not affect pro-IL-1β, pro-caspase-1, or ASC expression, suggesting that brief EtOH exposure inhibits NLRP3 inflammasome activation rather than the synthesis of its components [147]. Comparatively, chronic EtOH treatment upregulated NLRP3 expression and increased caspase-1 activation, as well as IL-1β and IL-18 release, in cultured murine cortical astrocytes [142]. These findings were corroborated in vivo, as chronic alcohol feeding upregulated NLRP3, ASC, pro-caspase-1, and pro-IL-1β expression, and increased caspase-1 activity, in the cortex and cerebellum of mice [126].

## 6. Emerging Connections Between Thiamine Deficiency and Neuroinflammation

EtOH does not have a singular target but rather acts on a number of neurochemical and physiological processes, thereby exhibiting a high level of promiscuity. This unique behavior has several implications for AUD, contributing to a range of pathomechanisms that influence disorder progression, including those which lead to metabolic dysfunction and neuroinflammation. However, instead of functioning as separate drivers of disease, these processes act synergistically. Therefore, rather than examine each in isolation, it is important to acknowledge the role they play collectively.

While a definitive relationship between TD and neuroinflammation has yet to be established, emerging evidence supports the coexistence of these two pathologies in AUD. In rats with TD, inflammatory genes and proteins associated with microglial activation were significantly upregulated in the thalamus and inferior colliculus [148,149]. Notably, Egr-1 and c-EBP-β, transcription factors implicated in inflammation following oxidative impairment, were also elevated in this model [148]. Consistent with these findings, treatment with pyrithiamine resulted in a 3-fold increase in IL-1β, 8-fold increase in TNF-α, and 26-fold increase in IL-6 levels in the thalamus of rats [150]. In another study exploring the impact of TD on microglial and astrocytic activation, TD was found to increase microglial CD40 and astrocytic CD40L, two proteins with regulatory roles in immune response [151]. Early deletion of CD40 decreased astrocytic CD40L levels and reduced neuronal death by 35%, whereas deleting CD40L decreased microglial CD40 levels and reduced neuronal death by 64%. Later removal had no protective effect, indicating that microglial and astrocytic interactions drive neuronal death in the early stages of TD. Furthermore, while M1 microglia are primarily dependent on glycolysis as an energy source, anti-inflammatory M2 microglia rely on fatty acid oxidation and oxidative phosphorylation [152]. Thus, it can be suggested that a deficiency in thiamine, which impairs aerobic cellular respiration by preventing the conversion of pyruvate to acetyl-CoA, could interfere with microglia adopting an anti-inflammatory phenotype.

There is also evidence for a relationship between TD and inflammatory signaling pathways involving TLR4 and NLRP3. In mice exposed to 12 days of a thiamine-deficient diet, increased TLR4 signaling was evident in the frontal cortex, with upregulation extending to the cerebellum after 16 days [153]. This highlights a positive association between the duration of TD and extent of neuroinflammation. A later study by the group reported elevated levels of cortical TLR4 and MyD88 in rats exposed to both chronic alcohol and TD [154]. Likewise, upregulation of the TLR4/MyD88 pathway was observed postmortem in the cortex and cerebellum of an alcohol-induced WE patient [154]. These findings are the first to establish a relationship between TLR4 and TD, suggesting that TLR4 inflammatory signaling is augmented by TD. In the first study to investigate NLRP3 signaling in TD, researchers noted marked activation of NLRP3 in the microglia of thiamine-deficient mice [155]. Overall, existing findings indicate that TD is related to a heightened state of TLR4- and NLRP3-related neuroinflammation.

Importantly, TD and neuroinflammation also contribute to disruptions in neurotransmission, further emphasizing how these systemic processes synergistically govern addiction. Foundational preclinical studies further demonstrate that TD enhances the rate of GABA synthesis, supporting a link between metabolic disruption and altered inhibitory signaling [156]. Glial cells play an important role in mediating this dysfunction. Thiamine-induced disruptions to cerebral metabolism contribute to microglial hyperactivation, which not only exacerbates neuroinflammatory responses but additionally upregulates GABAergic, glutamatergic, and dopaminergic transmission, potent drivers of addictive behaviors seen in AUD [149,157]. Increased GABAergic transmission in turn elicits the release of dopamine and endogenous opiates, neurotransmitters all strongly linked to reward responses that fuel addiction [39,158]. TD is further connected to glutamatergic dysfunction via astrocytic glutamate clearance. Post-mortem analysis of individuals with alcohol use disorder and confirmed Wernicke’s encephalopathy revealed decreased levels of astrocytic glutamate transporters in the frontal cortex, a change thought to contribute significantly to neuronal cell death in this region [159]. Altogether, these findings illustrate how TD and neuroinflammation form an interdependent network of metabolic, immune, and neurotransmitter disruptions that collectively drive the neurobiological underpinnings of AUD.

## 7. Future Directions and Research Opportunities

AUD is a complex condition, involving not only maladaptive neurocircuitry but also metabolic dysfunction and immune dysregulation. Current FDA-approved medications for AUD, which aim to regulate drinking behaviors, fail to address the broader physiological disturbances associated with problematic alcohol use. Therefore, novel therapeutic candidates for AUD should aim to not only moderate excessive alcohol consumption but to simultaneously address disease pathology at a systemic level. Although evidence linking TD and neuroinflammation in AUD remains largely preliminary, requiring further mechanistic and longitudinal investigation, their apparent interrelatedness underscores their potential as complementary therapeutic targets in AUD. Thiamine supplementation, for example, is recommended for individuals with AUD due to its cognitive benefits and is frequently administered to patients with WE in emergency settings [81,82]. However, despite this clinical utility, it remains largely underutilized in broader treatment paradigms for AUD. In pursuit of more comprehensive interventions, multiple therapeutic avenues can be considered, including the development of novel small molecules, evaluation of nutraceutical candidates, and identification of new molecular targets.

Given that a key driver of AUD pathophysiology is inflammatory signaling, small molecule inhibitors targeting TLR4 and the NLRP3 inflammasome represent promising therapeutic candidates for mitigating alcohol-induced neuroimmune activation and systemic inflammation. One example includes TAK-242, a small molecule which has demonstrated neuro- and hepatoprotective effects in preclinical models of ischemic stroke, traumatic brain injury, and inflammation-induced hepatic fibrosis [160,161,162]. The compound has also been investigated in the context of ethanol-induced immune activation, where pre-treatment of TAK-242 prior to binge EtOH in rats preserved synaptic plasticity in the hippocampus, especially notable as deficits in synaptic plasticity have been associated with the transition from substance misuse to dependence [163,164]. Similarly, MCC950, a selective NLRP3 inhibitor, has been demonstrated to not only mitigate neuroinflammatory pathology, but also lower alcohol consumption and preference [144,165]. More research is needed to comprehensively evaluate the potential benefit of these compounds.

In addition to small molecule inhibitors, nutraceuticals represent another promising class of interventions capable of both modulating EtOH consumption behavior and addressing the systemic consequences of alcohol consumption. One nutraceutical that directly addresses TD in AUD is benfotiamine, a derivative of thiamine with higher bioavailability that is emerging as an innovative treatment option for inflammation and oxidative stress in neurological conditions [155,166]. Benfotiamine administration was also found to reduce NLRP3-induced inflammation in LPS- and ATP-stimulated microglia by decreasing ROS levels and attenuating autophagic impairments [155]. Additionally, the antioxidant properties of benfotiamine protect not just the brain from damage but also the liver, normalizing AST and ALT levels in an acute alcohol intoxication rat model [167]. Clinical trial data indicate that benfotiamine significantly reduced alcohol consumption over time across all study participants, highlighting its capacity to treat thiamine deficiency, neuroinflammation, and factors contributing to AUD [168].

In parallel with the continued evaluation of existing compounds, the discovery of novel therapeutic targets offers an additional strategy to expand the current treatment landscape for AUD. An additional emerging target is triggering receptor expressed by myeloid cell 2 (TREM2), a macrophage membrane protein which functions as a negative regulator of inflammation, promoting phagocytosis of various inflammatory stimulators, including cellular debris and apoptotic neurons [169]. In the CNS, TREM2 signaling promotes microglial proliferation and lipid processing; accordingly, TREM2-deficient mice exhibit impaired myelin debris clearance and increased axonal damage following pharmacologically induced demyelination [170,171,172]. TREM2 is currently being explored as a pharmaceutical target in Alzheimer’s disease, via a novel high-affinity TREM2 antibody and small molecule agonist called VG-3927 [172,173]. However, recent studies assessing TREM2 in alcohol-induced neuroinflammation suggest a more complex and potentially context-dependent role, with evidence supporting both protective and detrimental microglial effects. In primary organotypic brain slice cultures, EtOH exposure reduced TREM2 expression and temporally increased TNF-α and IL-1β [174]. Similar reductions in TREM2 were observed in vivo, with binge-like EtOH exposure decreasing TREM2 expression by 28% in male and female 3xTg-AD mice, supporting a model of impaired anti-inflammatory microglial activity in AUD [175]. However, in a chronic EtOH consumption mouse model, increased TREM2 expression was observed and accompanied by reduced dendritic spine density and altered synaptic plasticity [176]. As these effects were reversed by TREM2 silencing, these findings implicate TREM2 in EtOH-induced circuit dysfunction. Given these disparate results, more research is required to determine the benefits of this potential target.

Taken together, exploring therapeutic innovations with diverse mechanisms of action might more effectively mitigate alcohol-related damage, while also addressing the psychological and behavioral factors that fuel AUD.

## 8. Conclusions

Given the multifactorial nature of AUD, effective therapeutic development poses a considerable challenge. To address this complexity, the implications of organ crosstalk, metabolic dysfunction, and inflammatory pathways involved in AUD must be further explored. Compelling evidence reveals that thiamine deficiency and neuroinflammation are integral components of AUD pathogenesis, representing distinct but interrelated biological factors which synergistically drive disease progression. Emerging evidence suggests that thiamine deficiency exacerbates neuroinflammation, altering microglial and astrocytic activation and impairing anti-inflammatory signaling. Additionally, thiamine deficiency appears to be implicated in the upregulation of TLR4 and NLRP3 pathways, further perpetuating cellular energy deficits and oxidative stress. Therefore, modulating related signaling pathways through the use of existing pharmacologic or nutraceutical agents may offer a viable strategy for functionally targeting AUD pathophysiology. In parallel, investigating novel molecular targets that regulate or intersect with these systems may expand future therapeutic options and improve treatment outcomes. Examining AUD in these contexts thus provides valuable insights into lesser explored mechanisms, highlighting areas requiring further inquiry, as well as promising directions for future research.

## Data Availability

No new data were created or analyzed in this study. Data sharing is not applicable to this article.

## Abbreviations

The following abbreviations are used in this manuscript:

AUD	alcohol use disorder
TD	thiamine deficiency
CNS	central nervous system
GABA	gamma-aminobutyric acid
LPS	lipopolysaccharide
TNF-α	tumor necrosis factor α
ALD	alcohol-associated liver disease
EtOH	ethanol
TPP	thiamine pyrophosphate
TK	transketolase
PDH	pyruvate dehydrogenase
ɑ-KG	alpha-ketoglutarate
ɑ-KGDH	alpha-ketoglutarate dehydrogenase
PPP	pentose phosphate pathway
TCA cycle	tricarboxylic acid cycle
NAD+	nicotinamide adenine dinucleotide
G6P	glucose 6-phosphate
TPK	thiamine diphosphokinase
ROS	reactive oxygen species
GSSG	glutathione disulfide
GSH	glutathione
BBB	blood–brain barrier
WKS	Wernicke–Korsakoff syndrome
WE	Wernicke’s encephalopathy
KS	Korsakoff syndrome
TLR4	toll-like receptor 4
PRR	pattern recognition receptor
PAMPs	pathogen-associated molecular patterns
DAMPs	damage-associated molecular patterns
TIR	Toll/IL-1 receptor
HMGB1	high-mobility group protein 1
MyD88	myeloid differentiation primary response gene 88
TIRAP	toll/interleukin-1 receptor domain-containing adaptor protein
NF- κB	nuclear factor-κB
TRIF	TIR domain-containing adaptor inducing IFN-beta
TRAM	TRIF-related adaptor molecule
HDACs	histone deacetylases
NOD	nucleotide oligomerization domain
NLRP3	NOD-like receptor protein 3
ASC	apoptosis-associated speck-like protein containing a CARD domain
AIM2	absent-in-melanoma 2
PYD	pyrin domain
NO	nitric oxide
TREM2	triggering receptor expressed by myeloid cell 2
ADH	alcohol dehydrogenase
ALDH2	aldehyde dehydrogenase 2
CYP2E1	cytochrome P450 2E1
